# Genetic screening for neovascular AMD: cost effective…not so quick

**DOI:** 10.1186/s40942-015-0024-5

**Published:** 2015-12-15

**Authors:** Elias Reichel

**Affiliations:** New England Eye Center Boston, 800 Washington St, Boston, MA 02111 USA

## Abstract

The following is a response to Brown and colleagues (Int J Retin Vitr 1:19, 2015), who analyzed the cost effectiveness of genetic screening for neovascular AMD in Category 3 (intermediate). As explained in this letter, it is premature to propose that genetic screening is cost effective in this setting. A simple clinical history and macular exam is highly cost effective and can easily guide screening strategies.

Brown and colleagues [1] have analyzed the cost effectiveness of genetic screening for neovascular AMD (NVAMD) in Category 3 (intermediate). Their analysis takes into account costs associated with genetic screening, follow-up and early treatment as well as patient value gain based upon QALY (quality-adjusted life-year) gain. Their results show that genetic testing may be cost effective if an incremental 4.5 % of NVAMD cases are identified early. I believe that this conclusion is flawed for two reasons.

First, the main premise of the paper is based upon the concept that models based on clinical history and phenotype are not as reliable as genetic models. However, there is strong evidence that the risk of progression to NVAMD can be assessed by clinical exam and history alone, and just as effectively as when genetic testing is incorporated into these models [2]. Therefore, the costs associated with genetic testing can be saved by using non-genetic models—a saving of two billion dollars that has yet to be incurred!

Second, Brown’s paper relies heavily on Yu et al.’s work [3] of models of genetic risk predicting progression to NVAMD. Genetic high risk Category 3 patients have a 26 % chance of developing NVAMD in 10 years; Category 2 patients have a 13 % chance of converting to NVAMD in 10 years. Because Brown’s work focuses on Category 3 solely, they ignore a substantially large number of patients who may want genetic testing and if so identified as being high risk genetically would require very long-term follow-up at presumably great expense. In other words, Category 2 patients are greater in number and when identified as being at high risk genetically will need frequent long-term follow-up. This paper does not consider this population of patients—who I suspect may want to know their genotype if genetic screening became readily available. This would substantially change the calculus of the cost-effectiveness of genetic screening—likely making it less cost effective. Simply following Category 2 clinically as they convert to Category 3 is highly cost effective without the need for genetic testing. For these reasons it is extremely premature to propose that genetic screening is cost effective—a simple clinical history and macular exam is highly cost effective and can easily guide screening strategies.

## References

[1] Brown GC, Brown MM, Lieske HB (2015). A value-based medicine cost-utility analysis of genetic testing for neovascular macular degeneration. Int J Retin Vitr.

[2] Chiu C-J, Mitchell P, Klein R (2014). A risk score for the prediction of advanced age-related macular degeneration. Ophthalmology.

[3] Yi Y, Reynolds R, Rosner B (2012). Prospective assessment of genetic effects on progression to different stages of age-related macular degeneration using multistate Markov models. Invest Ophthalmol Vis Sci.

